# Longitudinal brain health and neurological correlates of sexual strangulation in young adults: Study protocol for a prospective cohort study

**DOI:** 10.1371/journal.pone.0344672

**Published:** 2026-04-20

**Authors:** Keisuke Kawata, Claire V. Buddenbaum, Megan E. Huibregtse, Sage H. Sweeney, Hu Cheng, Molly Rosenberg, Debra Finn, Sharlene D. Newman, Grace M. Wetzel, Jenna M. Hohne, Kirstin M. West, Molly McGuire, Kate H. Miller, Gage Ellis, Emily F. Galper, John R. Feiner, Eve Valera, Debby Herbenick

**Affiliations:** 1 Department of Kinesiology, Indiana University School of Public Health-Bloomington, Bloomington, Indiana, United States of America; 2 Program in Neuroscience, The College of Arts and Sciences, Indiana University, Bloomington, Indiana, United States of America; 3 Department of Pediatrics, Indiana University School of Medicine, Indianapolis, Indiana, United States of America; 4 Department of Health and Kinesiology, University of Illinois Urbana-Champaign, Urbana, Illinois, United States of America; 5 Department of Psychological and Brain Sciences, College of Arts and Sciences, Indiana University, Bloomington, Indiana, United States of America; 6 Department of Epidemiology and Biostatistics, Indiana University School of Public Health-Bloomington, Bloomington, Indiana, United States of America; 7 Alabama Life Research Institute, University of Alabama, Tuscaloosa, Alabama, United States of America; 8 Department of Applied Health Science, Indiana University School of Public Health-Bloomington, Bloomington, Indiana, United States of America; 9 The Center for Sexual Health Promotion, Indiana University School of Public Health-Bloomington, Bloomington, Indiana, United States of America; 10 Department of Anesthesia and Perioperative Care, University of California at San Francisco School of Medicine, San Francisco, California, United States of America; 11 Department of Psychiatry, Harvard Medical School, Massachusetts General Hospital, Charlestown, Massachusetts, United States of America; PLOS: Public Library of Science, UNITED KINGDOM OF GREAT BRITAIN AND NORTHERN IRELAND

## Abstract

Sexual strangulation (also referred to as choking during sex) has become increasingly prevalent among young adults and disproportionately affects females. Epidemiological studies indicate that more than half of college-aged women report a lifetime history of being strangled during sex, and it is most often described as consensual. Despite its widespread occurrence, little is known about the acute and cumulative neurological consequences of this behavior. Pilot studies suggest associations between frequent sexual strangulation and elevations in blood biomarkers of neural injury, alterations in brain structure and connectivity, and increased mental health symptoms; however, existing evidence is limited by cross-sectional designs, short-term follow-ups, and small sample sizes. This prospective cohort study is designed to characterize both the acute and longitudinal neurological effects of sexual strangulation using multimodal assessments. A total of 200 young adult females (100 with frequent sexual strangulation exposure and 100 controls with no lifetime strangulation history), along with a pilot cohort of 40 males, will undergo comprehensive evaluations including blood-based biomarkers of neural injury and inflammation, multimodal MRI, cognitive and oculomotor testing, retinal imaging, and mental health assessments. Acute neurological responses will be assessed approximately 24 hours following a sexual event involving strangulation (or non-strangulation sex in controls), while longitudinal follow-up will occur every six months over 30 months to examine cumulative exposure effects. Semi-structured interviews conducted at each time point will characterize strangulation practices, contextual factors, consent dynamics, and associated symptoms. The primary objectives are to (1) examine baseline group differences in neurological health between individuals with frequent sexual strangulation exposure and non-strangulation controls, (2) identify acute neurological alterations following sexual strangulation, (3) determine associations between cumulative strangulation exposure and neural cellular, physiological, and functional integrity over time, and (4) characterize young adults’ experiences, contextual factors, and perceptions of sexual strangulation through semi-structured interviews. Collectively, this study aims to establish temporal relationships between sexual strangulation and neurological health outcomes and to inform clinical guidance, sexual health education, and harm-reduction strategies related to this increasingly common sexual practice.

## Introduction

Sexual strangulation, often referred to as choking during sex, has emerged as a common sexual practice among young adults [1–4]. Although frequently described as consensual, this behavior disproportionately affects females [5–7] and has become increasingly normalized through mainstream media, popular music, pornography, and social media [4,8–17]. Large-scale epidemiological surveys indicate that sexual strangulation is highly prevalent in college-aged populations, with more than half of young adult women reporting a lifetime history of being strangled during sex and a substantial proportion reporting repeated exposure [18,19]. Despite its widespread occurrence, sexual strangulation remains poorly understood from a neurological and physiological standpoint, and empirical data capable of informing clinical guidance or sexual health education are limited.

From a biological perspective, sexual strangulation involves external compression of the neck that can partially or fully obstruct cerebral blood flow, venous return, and/or airway passages [4,7]. These mechanisms can result in transient cerebral hypoxia and ischemia, physiological stress responses, and alterations in cerebral perfusion [20]. Acute symptoms commonly reported following strangulation include dizziness, visual disturbances, disorientation, and, in some cases, loss of consciousness [20]. Experimental and clinical literature from other contexts involving asphyxia or vascular compression, such as intimate partner violence, autoerotic asphyxiation, and certain martial arts practices, demonstrates that even brief episodes of cerebral hypoxia can disrupt neural cellular integrity, particularly in vulnerable brain regions such as the hippocampus and other subcortical structures [21–25].

Emerging pilot data suggest that sexual strangulation may be associated with both short-term and longer-term neurological alterations. Specifically, young adult females who have been strangled at least 4 times during sex in the past month showed higher levels of circulating S100B level [26], which is indicative of astroglial activation, increased cortical thickness in widespread brain regions [27], interhemispheric differences in neural activation patterns [28], and inefficient neural effort during working memory tasks compared to strangulation naïve controls [29]. Furthermore, a single event of being strangled during sex was also associated with acute impairments in ocular convergence, blunted positive effects of sexual activities on mental health [30], and elevations in several inflammatory cytokines and chemokines (e.g., CCL-2, VEGF-A) [31]. However, interpretation of these findings has been constrained by methodological limitations, including reliance on a cross-sectional design, smaller sample sizes, and limited ability to distinguish pre-existing differences from strangulation-related effects.

A critical gap is the absence of longitudinal, multimodal studies capable of characterizing neurological health at baseline, assessing acute responses following sexual strangulation, and tracking cumulative effects over time. Without such data, it remains unclear whether observed neurological differences reflect pre-existing vulnerabilities, transient brain responses, or progressive neural changes associated with repeated exposure. Additionally, little is known about how contextual variables, such as frequency, methods, perceived consent, and subjective experiences, may modify neurological outcomes.

To address these gaps, the present study employs a prospective cohort design integrating multimodal neuroimaging, blood-based biomarkers, cognitive and oculomotor assessments, retinal imaging, and semi-structured interviews. This approach is designed to (1) examine baseline neurological differences between individuals with frequent sexual strangulation exposure and non-strangulation controls, (2) characterize acute neurological responses following sexual strangulation, (3) evaluate associations between cumulative exposure and neural cellular, physiological, and functional integrity over time, and (4) characterize participants’ experiences, perceptions, and behaviors related to sexual strangulation. By establishing temporal relationships and integrating biological and behavioral data, this study aims to generate critical evidence to inform clinical awareness, sexual health education, and harm-reduction strategies related to this increasingly common sexual practice.

### Aims and hypothesis

The aim of this study is to characterize baseline, acute, and longitudinal brain health associated with sexual strangulation in young adults using multimodal neurobiological, cognitive, and behavioral assessments.

### Specific aims

- Aim 1 To examine potential group differences at baseline in neurological health between the strangulation and control groups through comprehensive neurological measures.
  - Hypotheses: (1a) Significant differences in brain activation patterns and neuronal connectivity, increases in blood biomarkers for astrogliosis, inflammation, and axonal microstructural injury, alteration in retinal integrity, and declines in cognitive, ocular-motor, and mental health function will be observed in the strangulation group compared to controls. (1b) We will explore potential mediating factors, including behavioral, history, and broader demographic factors, driving group differences in neurological health.
- Aim 2: To identify the acute neurological effects of sexual strangulation through the response profile of multimodal neuroimaging, blood biomarkers, cognitive and mental health assessments, and visual health assessments.
  - Hypotheses: (2a) Within the strangulation group, significant alterations in brain activation patterns and neuronal connectivity, increases in blood biomarkers for astrogliosis, inflammation, and axonal microstructural injury, alteration in retinal integrity, and declines in cognitive, ocular-motor, and mental health function will be observed acutely (~24h) after being strangled during sex compared with baseline. (2b) The control group will have no worsening of neurological outcomes, resulting in a significant group difference at a post-acute time point. Also, individual cerebral hemodynamics via perfusion measure and fMRI activation patterns in risk and reward pathways will be explored in response to acute sexual strangulation events.
- Aim 3 To determine the associations between cumulative exposure to sexual strangulation and neural cellular, physiological, and functional integrities.
  - Hypotheses: (3a) Frequent sexual strangulation will correlate with negative neurological outcomes, and this association will strengthen over time. (3b) The neurological impact of cumulative strangulation will be pronounced in blood biomarkers, indicative of astrogliosis, inflammation, and reduced axonal microstructural integrity, surface and subcortical morphology, neural activation patterns in the limbic regions, and cognitive function. (3c) Cumulative effects of sexual strangulation will also be captured through a series of mental health and visual health assessments. For those switching their sexual behavior (e.g., controls who report new onset of strangulation experiences), an exploration of how brain health changes as they initiate or cease engaging in sexual strangulation will be conducted.
- Aim 4: To examine longitudinal characteristics, perceptions, and experiences of sexual strangulation via semi-structured interviews.
  - Semi-structured interviews in connection with each laboratory visit will address how one’s experiences with sexual strangulation change over time in terms of frequency, duration, methods of strangulation, consenting status, motivations, and associated symptoms. These data will not only play a crucial role in informing behavioral targets for educational guidelines and interventions, but also identify behavioral patterns and contextual factors that modify neurological outcomes. For example, our exploratory analysis will examine the role of alteration in consciousness (AIC), such as visual changes, disorientation, loss of consciousness, or dizziness) on neurological outcomes by comparing AIC and non-AIC subgroups.
- Exploratory Aim 5: To examine neurological implications of sexual strangulation among a pilot cohort of males.
  - Since no neurological data related to sexual strangulation exist for males, pilot cohorts of males (n=20 per group) will be included to generate preliminary data and hypotheses for future research.
- Exploratory Aim 6: To explore various contextual and modulatory factors that are influencing neurological and psychological wellbeing over time in relation to sexual strangulation and non-strangulation related sex.
  - Leveraging comprehensive assessments of participants’ demographics, prior sexual history, and contextual factors (e.g., menstrual cycle, birth control, sleep, substance/tobacco/alcohol use), we will explore how these factors contribute to individual responses to sexual strangulation and general sexual activities. This aim will include exploration of those who switch sexual behavior (e.g., stopping strangulation or control starting to engage in strangulation).

### Outcome measures

Our outcomes (Table 1) will be assessed by comparing group differences, specifically group (strangulation vs control) x time interaction, as well as within-group time-course changes in outcomes.

**Table 1:** Outcome measures.

Outcome Measure	Type	Test/Task and Description
Blood biomarkers	Primary outcome	The primary outcome analyses will be comparing group differences (group x time interactions) and within-group time-course changes in blood biomarkers, specifically NF-L, tau, GFAP, UCH-L1, p-tau 181, p-tau 217, and S100-beta. Additional panel of inflammatory cytokines and chemokines will be explored: IL-1b,1a,2,6,8,10,17; CCL2,3,5; and VCAM, PCAM, angiopoietin, and neureglin.
Structural neuroimaging	Primary outcome	Cortical and subcortical morphometry analyses will yield macro-structural variables (thickness, volume, gyrification, sulcal depth) across the brain. For analysis of microstructural integrity, DTI and NODDI measures will be used to derive MD, axial and radial diffusivity, fractional anisotropy, NDI, ODI, and ICVF.
fMRI imaging	Primary outcome	Resting-state connectivity will be examined throughout the whole brain and specific seeded regions including the DLPFC, angular gyrus, and cingulate gyrus. Task-based fMRI using the BART will assess neural activity (inhibition and excitation) during risk-taking behaviors in the risk-reward circuitry.
Clinical assessments	Primary outcome	Cognitive function will be measured using the NIH Toolbox Cognition Battery, which examines 5 cognitive domains (executive function, episodic memory, language, working memory, and processing speed). Oculomotor function will be tested by NPC. The NPC measures will be repeated twice, and the mean NPC (in centimeters) will be used for analyses. Mental health-related symptoms will also be treated as primary outcomes, which include depression, stress, anxiety, ADHD, personality traits, and sleep.
Retinal health	Secondary outcome	Optical coherence tomography/angiography (OCT/A) will be used to assess retinal neural structure. The primary OCT variables will be macula CSF thickness (an indicator of the gain or loss of neurons or glia in the inner nuclear, ganglion cell and nerve fiber layer), and cup-to-disc ratio (an indicator of neurodegeneration at the optic disc). Retinal vascular structure will be acquired using the OCT angiography. The primary OCT/A variable examined will be FAZ area reflecting the size of the central portion of the macula which contains no blood vessels, and which increases in size with loss of capillaries in the surrounding region).
Quantitative susceptibility mapping (QSM)	Secondary outcome	Quantitative Susceptibility Mapping of whole brain, as well as regions of interest analysis, will be conducted to inspect the tissue and brain architectural response to sexual strangulation.
Semi-structured interview	Secondary outcome	The interview will assess participants’ mood, relationship status, relationship quality, sexual agency, and overall sexual behaviors. The interview will also characterize strangulation-related characteristics (e.g., onset, frequency, duration; strangulation method (e.g., both hands, ligature); and responses from being strangled (e.g., visual changes, dizziness, disorientation, loss of consciousness), which will be asked in a 5-point Likert scale (0 = none, 5 = severe), as well as their levels of enjoyment and pleasure. The study will explore various dimensions of consent, including the timing and frequency of consent, who initiates consent discussions, and whether the act has ever become uncomfortable despite initial consent.

Note: NF-L (neurofilament light), GFAP (glial fibrillary acidic protein), UCH-L1 (ubiquitin C-terminal hydrolase-L1), IL (Interleukin), CCL (C-C motif chemokine ligand), VCAM (vascular cell adhesion molecule), PCAM (Platelet endothelial cell adhesion molecule), DTI (diffusion tensor imaging), NODDI (neurite orientation dispersion and density imaging), MD (mean diffusivity), NDI (neurite density index), ODI (orientation dispersion index), ICVF (intracellular volume fraction), DLPFC (dorsolateral prefrontal cortex), BART (balloon analogue risk task), NPC (near point of convergence), ADHD (attention deficit/hyperactivity disorder), OCT/A (Optical coherence tomography/angiography), FAZ (foveal avascular zone)

## Methods

### Participants

This prospective cohort study will enroll a total of 240 young adults, including 200 females and a pilot cohort of 40 males. Female participants will be assigned to one of two primary groups: individuals with frequent exposure (≥4 times per month) to sexual strangulation (n = 100) and age-matched controls with no lifetime history of being strangled during sex (n = 100). An additional pilot cohort of males (n = 20 per group) will be recruited to generate preliminary data on neurological correlates of sexual strangulation in males. The recruitment is anticipated to start in late February 2026 until March 2029, and all data collection is expected to finish by May 2030. Primary analyses will finish by May 2031. Eligible participants will be between 18 and 26 years of age and recruited primarily from the Indiana University campuses and surrounding community. Longitudinal follow-up of enrolled participants will extend up to 30 months. All participants will provide electronic written informed consent prior to enrollment. The initial study protocol has been approved by the Indiana University Institutional Review Board (#27400) on Jun 04, 2025. Upon completion of primary and secondary analyses, all relevant data will be made publicly available in sites including *brainlife* and inter-university Consortium for Political and Social Research (ICPSR).

Inclusion and exclusion criteria are summarized in Table 2. For the strangulation group, inclusion requires self-report of being strangled during sex at least four times in the month prior to enrollment, which will be verified during a screening interview. Control participants must report no lifetime history of sexual strangulation. Exclusion criteria are limited to conditions necessary for participant safety or study feasibility and include head or neck injury in the past 6 months including concussions, current diagnosis of psychosis, schizophrenia, and seizure disorder, currently taking anti-psychotic medicine, history of brain aneurysm or brain tumor, and current pregnancy. Common psychiatric conditions prevalent in young adults, including depression, anxiety disorders, bipolar disorder, and attention-deficit/hyperactivity disorder (ADHD), will not be excluded and will be assessed and accounted for analytically. Additionally, participants with implanted metal/electro/magnetic devices will not undergo MRI data collection, but they will complete all other assessments; thus, not excluded from the study.

**Table 2:** Inclusion and exclusion criteria.

**Inclusion Criteria**
• 18–26 years of age
• Being strangled during sex, on average, three times in the past month (strangulation group)
• Age-matched participants with no lifetime experience of being strangled during sex (control group).
**Exclusion Criteria**
• Currently pregnant
• Have had a head or neck injury within the past 6 months
• Current diagnosis of psychosis, schizophrenia, and seizure disorders
• Currently taking anti-psychotic medicine
• History of brain aneurysm or brain tumor

**Fig 1:**
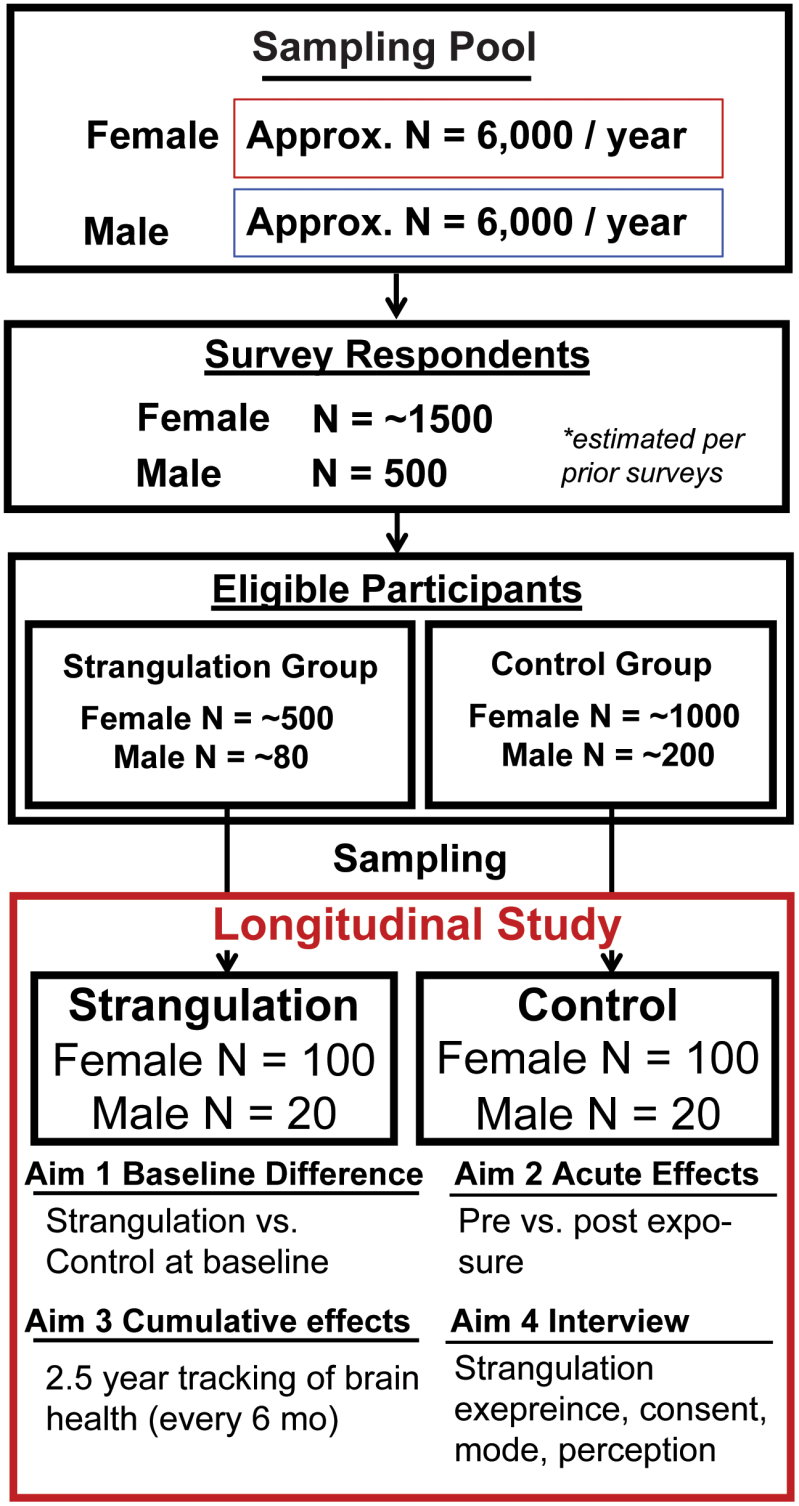
Schematic study design.

Participants may be withdrawn from the study without their consent if they become ineligible during the study period, exhibit intoxication at study visits, or engage in behavior that compromises participant or staff safety. Participants who change sexual behavior during the study (e.g., controls who begin engaging in sexual strangulation or strangulation participants who discontinue the behavior) will continue follow-up when feasible, with data contributing to primary analyses up to the point of behavioral change and exploratory analyses thereafter. Table 3 indicates the status of enrollment and Fig 1 depicts the study flow.

**Table 3:** SPIRIT checklist, February 2026.

	STUDY PERIOD						
	Enrolment	Allocation	Post-allocation				Close-out
**TIME POINT**	***-t***_***1***_	**0**	***t***_***1***_	***t***_***2***_	***t***_***3***_	***t***_***4***_	***t***_***x***_
**ENROLLMENT:**	0						
**Eligibility screen**	0						
**Informed consent**	0						
**Allocation**		0					
**ASSESSMENTS:**							
***[Demographics, Questionnaire, SCID-5, interview]***		0					

### Study procedures

#### Study design overview.

This study employs a mixed-methods prospective cohort design integrating multimodal neurological assessments and semi-structured interviews (Fig 1). Participants will be enrolled into either a sexual strangulation group or a non-strangulation control group based on screening criteria and will undergo baseline neurological and behavioral assessments. Acute neurological responses will be evaluated following a sexual event involving strangulation (or non-strangulation sex in controls), and longitudinal follow-up assessments will be conducted every six months for up to 30 months to characterize cumulative exposure effects and temporal changes in neurological health.

#### Screening and enrollment.

Potential participants will be identified primarily through a campus-wide probability survey focused on sexual health and behavior that is administered during the first two to three years of the study, supplemented by community-based recruitment strategies, including university’s classified sites and social media platforms. Individuals who indicate willingness to be contacted for follow-up research will be randomly stratified (via Randomization function within *R*), with first- and second-year students taking the priority spots to accommodate those who are likely be in school throughout the entirety of 30 months follow-up. Invited participants will complete a prescreening questionnaire to confirm eligibility criteria, including age, sexual strangulation history, and key exclusionary medical or neurological conditions. Eligible individuals will be invited to a virtual consent meeting, during which electronic written consent will be obtained. Following the virtual consent meeting, participants will undergo an additional virtual meeting, and research staff will administer QuickSCID-5 (Quick Structured Clinical Interview for DSM-5 Disorders) to assess current and history of mental health diagnosis. When participants endorse any symptoms related to psychosis and/or schizophrenia, research staff will then conduct a SCID-5 Research Version – Psychotic module B/C to determine the eligibility of participants [32]. During the virtual meeting, research staff will also administer The Alcohol, Smoking and Substance Involvement Screening Test (ASSIST) [33]. When participants meet the inclusion criteria and are free of exclusionary criteria, they will be enrolled in the study and assigned a study-specific ID. Prior to baseline visit, participants are asked to abstain from the following behaviors and caffeine: abstain from sex involving choking and any activities involving pressing or squeezing of the neck (e.g., martial arts chokeholds) 48 hours prior to lab visit, no strenuous exercise (e.g., sports games, cross-fit) 24 hours prior to the visits, no mild to moderate exercise on the day of lab visit, no sexual activities on the day of lab visit, and no caffeine on the day of lab visit.

#### Baseline assessment.

Between enrollment and the baseline laboratory visit, participants will complete a series of online questionnaires assessing demographics, medical and neurological history, sexual behavior history, and Adverse Childhood Experiences (ACES). At the baseline visit, participants will undergo various assessments, including testing day questionnaires to gauge various contextual information (menstrual cycle, exercise), capillary blood sampling to test blood fatty acid components, urinalysis to verify pregnancy status, blood sampling for biomarker analysis, MRI, cognitive testing, oculomotor assessments, retinal imaging, and mental health questionnaires. Baseline assessments are designed to characterize neurological health prior to acute or longitudinal exposure assessment and to allow examination of baseline group differences between participants with frequent sexual strangulation exposure and non-strangulation controls. The baseline visit will end with setting up a MyCap account within Research Electronic Data Capture (REDCap) for participants for acute visit purposes. Participants in both the strangulation group and control group will participate in the acute aim (aim 2), but control participants who are not sexually active will not be participating in the acute aim.

#### Acute assessment following sexual activity.

Participants eligible for the acute aim will be monitored following baseline assessment using brief daily electronic surveys to determine the occurrence of sexual activity and the presence or absence of strangulation. For participants in the strangulation group, an acute laboratory visit will be scheduled within approximately 24 hours following a sexual event involving strangulation. For control participants, an acute visit will be scheduled within approximately 24 hours following a sexual event without strangulation. Participants who do not engage in sexual activity during the monitoring period will remain eligible for longitudinal follow-up but may not contribute to acute analyses. The acute visit will replicate key components of the baseline assessment, including blood collection, neuroimaging, cognitive testing, oculomotor and retinal assessments, and mental health questionnaires. These data will be used to characterize short-term neurological responses associated with sexual strangulation relative to baseline and control conditions.

#### Longitudinal follow-up.

All participants will enter a longitudinal follow-up phase consisting of laboratory visits every six months for up to 30 months following baseline assessment. Each follow-up visit will include repeat multimodal neurological assessments identical to baseline measures, allowing evaluation of changes in neural cellular, physiological, and functional integrity over time. Longitudinal assessments are designed to capture cumulative exposure effects and temporal trajectories of neurological health associated with sexual strangulation. Participants who change sexual behavior during the study (e.g., controls initiating sexual strangulation or strangulation participants discontinuing the behavior) will continue follow-up when feasible. Data collected prior to behavioral change will contribute to primary analyses, while post-change data will be used in exploratory analyses to examine within-individual changes.

#### Semi-structured interviews.

Semi-structured interviews will be conducted at baseline (within 1 week from the baseline neurological assessments), following acute laboratory visits (within 72 hours), and after each longitudinal follow-up visit (within 1 week from the baseline neurological assessments). Interviews will assess participants’ experiences with sexual strangulation, including frequency, duration, methods, perceived consent, contextual factors, associated symptoms, and changes in behavior over time. These interviews will provide behavioral and contextual data to complement neurobiological findings and to inform interpretation of neurological outcomes.

### Measurements

#### Blood fatty acid composition.

At baseline and all follow-up timepoints, a capillary blood sample from a fingertip will be collected and sent to OmegaQuant Analytics for blood fatty acids composition analysis.

#### Psychiatric and mental health questionnaires.

The following questionnaires will be used to gauge symptoms related to various psychiatric and mental health conditions, including Patient Health Questionnaire-9 (PHQ-9) for depression, Generalized Anxiety Disorder-7 (GAD-7) for anxiety, Perceived Stress Scale (PSS) for perceived stress, adult ADHD self-report scale (ASRS) for ADHD-related symptoms, Multidimensional Personality Questionnaire (MPQ) for personality traits, Extended–Hurt, Insulted, Threaten, Scream (E-HITS) for history of domestic/intimate partner violence with an additional question regarding strangulation experience, Penn State Electronic Cigarette Dependence Index (PS-ECDI) for e-cigarette use, Pittsburgh Sleep Quality Index (PSQI) for sleep, Migraine Disability Assessment (MIDAS) for migraine, Life Events Checklist-5 (LEC-5) to screen for lifetime traumatic events, and PTSD Checklist for DSM-5 (PCL-5) to assesses symptoms of PTSD.

#### Blood biomarker assessment.

Capillary blood sampling using the Tasso blood collection device will be performed each test session. A trained phlebotomist will thoroughly clean the area with an alcohol swab and collect two sets of 400 μL of capillary blood sample using the Tasso device. Plasma levels of NF-L, Tau, UCH-L1, GFAP, p-tau 181, and p-tau 217 will be assessed by Quanterix’s Simoa SR-X assays. Various cytokines and chemokine markers, including S100B, IL-1b,1a,2,6,8,10,17; CCL2,3,5,11; and VCAM, PCAM, angiopoietin, and neuregulin, will be assessed by custom Luminex panels.

#### Neuroimaging assessment.

Neuroimaging assessments will take place in the Indiana University’s Imaging Research Facility (IRF), which houses a research-dedicated 3T Siemens Prisma MRI scanner equipped with a 64-channel head/neck coil.

High-resolution anatomical images (T1), which will be used for morphometry analyses, will be acquired using 3D MPRAGE pulse sequence: TR/TE = 2400/2.3ms, TI = 1060ms, flip angle = 8, field-of-view = 256 mm, matrix = 320x320, bandwidth = 160 Hz/pixel, iPAT = 2, resulting in 0.8 mm isotropic resolution. Both resting state fMRI (rs-fMRI) and task-based fMRI data will be collected using an SMS single-shot EPI sequence: FOV = 216 mm, TR/TE = 800/30 ms, flip angle = 52°, matrix = 90 × 90, resolution = 2.4 mm isotropic, and multiband acceleration factor = 6, with 100 volumes for rs-fMRI and 240 volumes for task-based fMRI. The rs-fMRI data will be acquired while the participant relaxes with eyes open and passively views a crosshair for 12 minutes. For task-based fMRI, the BART task [34], which has two, 8-min blocks, will be administered. As participants continue to inflate a balloon for greater reward, the probability of an explosion increases to mimic real-world risk-reward decision making. For each trial, the screen will display the image of a purple balloon above a small green rectangle, which indicates that she/he needs to make a decision (inflate or win). Participants will accumulate their win/wager throughout the two, 8-min block, experiments. Potential regions of interest (ROI) include the dorsal anterior cingulate cortex (dACC), nucleus accumbens (NAc), and insula due to their involvement in reward and decision-making processes [35]. Our DTI protocol is adapted from the Adolescent Brain Cognitive Development study [36], which is optimized for NODDI analysis. Two consecutive DTI sessions with opposite phase encoding directions will be performed with a simultaneous multi-slice (SMS) single-shot spin-echo echo-planar imaging (EPI) pulse sequence (TE = 89.4ms; TR = 3590s, 1.5 mm isotropic resolution). Each session has 80 images with different diffusion weightings and gradient directions (7 b = 0 s/mm2, 6 directions with b = 500 s/mm2, 15 directions with b = 1000 s/mm2, 15 directions with b = 2000 s/mm2, and 60 directions b = 2500 s/mm2). The total scan time is 10 minutes. Quantitative Susceptibility Mapping (QSM) will be collected using a three-dimensional flow-compensated multiecho gradient-echo sequence with a repetition time of 45 msec, a total of five echoes (time of the first echo, 13 msec) with echo spacing of 6 msec, flip angle of 20°, field of view of 24 × 24 cm, matrix of 512 × 256, and 88 sections acquired with 1.5-mm thickness.

#### Cognitive function.

Cognition will be measured using the NIH Toolbox Cognition Battery [37]. Multiple forms will be used to minimize practice effects. This battery consists of 7 instruments testing 5 cognitive domains: 1. Dimensional Change Card Sort Test (cognitive flexibility); 2. Flanker Inhibition Test (inhibitory control); 3. Picture Sequence Memory Test (episodic memory); 4. Picture Vocabulary Test (language); 5. Oral Reading Recognition Test (language); 6. Sorting Working Memory Test (working memory); and 7. Pattern Comparison Processing Speed Test (processing speed). The battery takes 20 minutes to complete on a tablet.

#### Near point convergence (NPC).

NPC will be measured based on our established protocol [38–42]. Using the accommodative ruler, a target (14-point letter) will be moved toward the eyes at a rate of 1–2 cm/s. NPC will be recorded when participants report diplopia has occurred, or the tester observes eye misalignment. The assessment will be repeated twice, and the mean NPC value will be used for analyses.

#### Optical coherence tomography/angiography (OCT/A).

Retinal neural structure will be imaged using high-definition spectral domain OCT, which will be acquired using a Zeiss Cirrus 5000 OCT scanner. The OCT variables examined will be macula CSF thickness (an indicator of the gain or loss of neurons or glia in the inner nuclear, ganglion cell and nerve fiber layer), and cup-to-disc ratio (an indicator of neurodegeneration at the optic disc). Retinal vascular structure will be acquired using the OCT angiography (OCT/A) capability of the Cirrus 5000. The primary OCT/A variable examined will be foveal avascular zone (FAZ) area reflecting the size of the central portion of the macula which contains no blood vessels, and which increases in size with loss of capillaries in the surrounding region).

#### Semi-structured Interviews.

The interview will assess participants’ relationship status, relationship quality, sexual agency, and overall sexual experiences, including partnered strangulation. For participants in the strangulation group, we will assess strangulation-related characteristics (onset, frequency, duration, single vs. repetitive, reasons for continued engagement, perceived prevalence among their peers); strangulation method (e.g., both hands, ligature); and responses from being strangled (e.g., visual changes, dizziness, disorientation, loss of consciousness, neck pain, bruising, euphoric sensations, as well as their levels of enjoyment and pleasure. The interview will also include questions related to less common “rough sex” behaviors that may be associated with neurological sequelae (e.g., face/head slapping, smothering, gagging). Participants will be asked about contextual issues relevant to their sexual experiences, which may include arousal, orgasm, intimacy, and/or trust. We will transcribe and code the interview data. The study will explore various dimensions of consent, including the timing and frequency of consent, who initiates consent discussions, and whether the act has ever become uncomfortable despite initial consent. If discomfort has occurred, participants will be asked whether they have discussed these experiences with their partner. Interviews will be audio-recorded and transcribed, with the audio files then deleted. Interview data will be coded and analyzed following a thematic analysis approach consisting of five steps following Nowell et al. [43]

### Ethics and dissemination

The study will be conducted in accordance with the ethical principles outlined in the Declaration of Helsinki, 1996. This research was approved by the Indiana University Institutional Review Board (IRB #27400). Data will be collected using REDCap spreadsheet to organize data and store in a secured database until statistical analysis is conducted. The data will be stored indefinitely for data quality purpose.

### Safety considerations and adverse events

There will be an independent safety monitor who will monitor adverse events data, oversee procedures designed to protect the privacy of participants, and report any adverse event. Data and safety monitoring will occur annually. In the event of an adverse event, the safety monitor will prepare and submit a report to the IRB and will communicate between the team and IRB in any necessary investigation.

### Statistical analyses

#### Baseline group analysis.

To test whether neurological outcomes differ at baseline between the strangulation and control groups, we will use multivariable regression models with each baseline neurological outcome as the dependent variable and group (strangulation vs control) as the primary independent variable. For continuous outcomes (e.g., biomarker concentrations, imaging metrics, cognitive scores, NPC distance, OCT measures), we will use linear regression (or generalized linear models with appropriate functions if distributional assumptions are not met). For binary outcomes (e.g., presence/absence of symptoms or threshold-defined impairment), we will use logistic regression. Models will adjust for prespecified baseline covariates that are clinically relevant and/or imbalanced between groups, including age, BMI, prior concussion history, and baseline mental health symptoms. Because baseline neural health may be influenced by contextual factors, we will additionally consider time since last sexual event, substance use, sleep, and ovarian hormone levels/menstrual cycle phase as covariates when relevant to specific outcomes. For imaging outcomes, scanner-related and acquisition covariates (e.g., motion indices) will be included as standard quality-control adjustments. We will control for multiple comparisons using a false discovery rate (FDR) approach within each modality (biomarkers, imaging, ocular/retinal, cognition, mental health). As a complementary approach that leverages correlation among outcomes, we will conduct multivariate testing within domains (e.g., principal-component-based composite scores) to evaluate whether a domain shows an overall baseline difference by group prior to interpreting individual endpoints. Where appropriate, we also may consider exploratory mediation and explanatory models to help explain baseline group differences. We will use mediation analyses to estimate indirect effects while adjusting for confounders.

#### Acute effects.

To examine the acute neurological effects of sexual strangulation, we will use a series of generalized linear mixed models (GLMMs) (or linear mixed-effects models for normally distributed outcomes) with each neurological variable as the dependent variable and visit type (baseline vs. post-acute) as the primary within-person predictor. Models will include a participant-specific random intercept to account for repeated measures. The primary interest will be the group (strangulation vs. control) × time (baseline vs. post-acute) interaction. Within-group time-effects will also be examined to assess group-specific time trends. Prespecified covariates will consider age, prior concussion history, and mental health diagnosis and/or mental health symptoms, as well as potentially adjusting for time since the most recent sexual event. We will also explore by including menstrual cycle phase and/or ovarian hormone levels as covariates. For neuroimaging outcomes, standard acquisition/quality covariates (e.g., motion indices) will be included. To address multiple comparisons across numerous endpoints, FDR will be applied within modality (biomarkers, imaging, ocular/retinal, cognition/mental health).

#### Cumulative effects.

To determine whether cumulative exposure to sexual strangulation is associated with longitudinal changes in neurological health, we will model repeated neurological outcomes collected every 6 months over 30 months using GLMMs (or linear mixed-effects models for continuous outcomes), a similar model structure to the acute aim. Each model will include a participant-specific random intercept to account for within-person correlation. Time will be modeled as a categorical variable (baseline, 6, 12, 18, 24, 30 months) for primary analyses to allow non-linear trajectories, and as a continuous variable in sensitivity analyses.

Primary longitudinal analysis will focus on: (1) overall group differences in trajectories between the strangulation and control groups via group × time interaction, and (2) within-group change over time (especially within the strangulation group) to identify when divergence from baseline emerges. Covariates will include age, prior concussion history, mental health diagnosis, and other clinically relevant time-varying factors assessed at each visit (e.g., mental health symptoms, substance use). For imaging outcomes, motion indices will be included.

To directly test the exposure-response hypotheses, we will incorporate time-varying measures of sexual strangulation exposure (e.g., frequency since last visit and cumulative frequency across follow-up; and, where available, intensity/duration and presence of alteration in consciousness) as predictors of neurological outcomes within the strangulation group. These models will estimate whether higher exposure is associated with worse neurological outcomes and whether this association strengthens over time by including exposure × time terms when appropriate.

#### Switching behavior.

During follow-up, some participants may initiate or discontinue sexual strangulation. We will conduct exploratory analyses among “switchers” using (a) time to initiation/cessation as an “intervention” and (b) within-person pre/post comparisons using mixed models with an indicator for post-switch period. These analyses will be labeled exploratory and interpreted as hypothesis-generating.

#### Semi-structured interview.

Interview data will be coded and analyzed following a thematic analysis approach consisting of five steps following Nowell et al. [43] Our analyses will be guided by the following questions: 1. What styles of strangulation do participants report in terms of neck placement, intensity, and being strangled once vs repetitively in a typical encounter? 2. How does one’s experiences with sexual strangulation change over time in the context of frequency, duration, intensity, and methods that they use? 3. What are the signs and symptoms related to sexual strangulation and to what extent do they change over time? 4. Why do people continue to engage in strangulation or decide to stop? As our earlier interview data were collected from only a single time point, it is critical to understand engagement in sexual strangulation prospectively and to examine change over time. Although these data will be novel and extend the literature on their own, they will also support interpretation of the neurological outcomes. For example, motives of engagement whether it is enjoyable/wanted or simply to please their partner may modulate neurological changes over time.

### Sample size

Sample size estimates were based on power calculations using pilot data and related literature. Pilot data demonstrated moderate to large effect sizes across blood biomarkers (Cohen’s d = 0.52–0.87), resting-state functional connectivity (d ≥ 1.2), and cortical morphology (d = 1.4–2.9). Using a conservative estimate of a moderate effect size (d = 0.60), an alpha level of 0.01 to account for multiple models, and a repeated-measures design, a sample of 60 females per group will provide 80% power to detect within-group acute changes and group-by-time differences. An additional pilot sample of 10 males per group will be included to generate preliminary effect-size estimates. For the cumulative longitudinal analyses, power calculations were informed by pilot data and evidence from related hypoxic and subconcussive injury models demonstrating moderate to large effects. Assuming a conservative effect size of d = 0.50, an alpha level of 0.01, and a worst-case dropout or group-switching rate of 20%, a total of 200 females (n = 100 per group) will provide greater than 80% power to detect group differences and longitudinal exposure-response effects over 30 months. To support hypothesis generation for future studies, an additional pilot cohort of 20 males per group will be included.

## Discussion

Sexual strangulation represents a distinct and understudied form of neurological stress that differs fundamentally from mechanically induced brain injury. Rather than direct impact or acceleration-deceleration forces, strangulation primarily induces transient cerebral hypoperfusion, hypoxia, and subsequent reperfusion, processes known to disrupt neural homeostasis and trigger neuroinflammatory cascades [44,45]. Evidence from preclinical hypoxia-ischemia models and clinical contexts such as non-fatal strangulation and autoerotic asphyxiation indicates that even brief interruptions of cerebral blood flow can elicit axonal stress, astrocyte activation, vascular dysregulation, and blood-brain barrier perturbation [22,46–50]. However, the extent to which these mechanisms operate in the context of consensual sexual strangulation, and whether they manifest as transient, compensatory, or cumulative neurological changes, remains unclear.

Blood-based biomarkers provide a sensitive and temporally dynamic index of neural cellular stress across a range of neurological conditions. Markers such as NF-L, tau, p-tau 181, and p-tau 217 reflect axonal cytoskeletal disruption, whereas GFAP and S100B index astrocytic activation and blood-brain barrier integrity, and UCH-L1 reflects neuronal cell body stress [51–53]. Prior work from our group has demonstrated elevations in subsets of these biomarkers among individuals with recent or frequent exposure to sexual strangulation, as well as acute increases in subset of inflammatory cytokines and chemokines (e.g., CCL2, VEGF-A) following strangulation-involved sexual events [31]. These findings are consistent with the hypothesis that strangulation elicits subtle but detectable axonal and glial responses, potentially driven by hypoxic-ischemic stress and inflammatory signaling. The present study will extend this work by assaying a panel of biomarkers that reflect complementary cellular processes. Thus, this study will help clarify whether sexual strangulation preferentially impacts specific neural pathways (e.g., astrocytic or axonal) or produces broader, system-level neurobiological disruption.

Neuroimaging provides critical insight into the structural and functional correlates of sexual strangulation. Pilot studies from our group have identified alterations in cortical morphology, interhemispheric imbalance in resting-state neural activity, and changes in functional activation during working memory tasks among individuals with frequent sexual strangulation exposure [27–29]. These findings suggest the possibility of compensatory neuroplastic responses to repeated hypoxic stress, particularly in cortical and limbic regions involved in cognition, sensorimotor integration, and mental health regulations. However, cross-sectional designs cannot determine whether such patterns reflect pre-existing vulnerability, adaptive responses, or progressive neural change. By integrating diffusion-based microstructural metrics, resting-state and task-based functional connectivity, and QSM across baseline, acute, and longitudinal time points, the proposed study is uniquely positioned to disentangle these possibilities.

Beyond neurobiological measures, sexual strangulation may influence cognitive, visual, and mental health functioning through mechanisms involving hypoxia, autonomic dysregulation, and psychological stress [7,19,54]. Acute hypoxic events have been shown to disrupt ocular-motor control, retinal integrity, attention, memory, and mood, while repeated exposure may contribute to longer-term symptom burden [55–57]. Our pilot study has demonstrated acute changes in NPC, alterations in mood-related outcomes, and increased reporting of neurological symptoms following strangulation-involved sex. [30] The integration of cognitive testing, ocular-motor and retinal assessments, and validated mental health measures in the present study will allow examination of whether neurobiological alterations are accompanied by clinically meaningful functional changes.

A key strength of the proposed study is its prospective, longitudinal design combined with semi-structured interviews that capture the contextual and experiential dimensions of sexual strangulation. Sexual strangulation occurs across a spectrum of motivations, intensities, and consent dynamics, and these factors may modify neurological risk. By characterizing frequency, methods, perceived consent, AIC, and associated symptoms, this study will help identify behavioral patterns that may amplify or mitigate neurological effects. While the observational design precludes causal inference, the temporal resolution afforded by acute and repeated follow-up assessments strengthens inference regarding exposure-response relationships.

Collectively, this study is designed to move the field beyond cross-sectional observation toward a temporally informed understanding of the neurological correlates of sexual strangulation. By integrating multimodal neurobiological, cognitive, visual, and behavioral data, the findings are expected to inform clinical awareness, guide future mechanistic and interventional research, and provide empirical evidence needed to support sexual health education and harm-reduction strategies. As sexual strangulation is now prevalent among young adults, rigorous scientific evaluation of its potential neurological consequences is essential to inform evidence-based guidance, screening, and prevention efforts.

### Patient and Public Involvement

It was not appropriate or possible to involve patients or the public in the design, or conduct, or reporting, or dissemination plans of our research.
